# The Impact of Digital Currency Innovation: Risk Spillover Effects Between the Cryptocurrency and Traditional Financial Markets

**DOI:** 10.3390/e28090949

**Published:** 2026-08-24

**Authors:** Lei Zhuang, Yang Liu

**Affiliations:** School of Economics and Management, Nanjing Tech University, Nanjing 211816, China

**Keywords:** digital currency, cryptocurrency, traditional financial markets, risk spillover, dynamic correlation, extreme market conditions

## Abstract

The rapid expansion of the digital currency market and the growing role of stablecoins as potential intermediaries have brought its interconnectedness with traditional financial markets to the forefront of global financial research. Using daily data from 4 January 2021 to 30 September 2025, this study constructs a variable system with the price indices of USDT and USDC as core digital currency proxies, alongside traditional financial asset indices for stocks, bonds, and gold derived via the entropy weight method. We employ a comprehensive set of econometric techniques, including static correlation analysis, vector autoregression (VAR), impulse response functions, and extreme-event shock tests, to systematically investigate the interdependence structure, risk spillover dynamics, time-varying co-movements, and structural changes between the two markets during extreme risk episodes. The findings reveal an overall weak and asymmetric bidirectional spillover relationship between the cryptocurrency and traditional financial markets. Volatility in the digital currency market is found to be largely endogenous, with a limited capacity to transmit shocks externally. Conversely, traditional financial markets—particularly the equity market—exert a more pronounced influence on the digital currency market. Critically, under the impact of extreme risk events, the cross-market linkages exhibit structural breaks; the direction and intensity of correlation can strengthen significantly or even reverse, demonstrating a clear state-dependency. This research provides empirical evidence for understanding the functional role of digital assets within the macro-financial system, their risk transmission pathways, and their implications for systemic financial stability. The findings offer valuable theoretical and practical insights for financial regulators in designing robust cross-market risk prevention frameworks and for investors seeking to optimize asset allocation strategies.

## 1. Introduction

### 1.1. Research Background and Significance

The proliferation of cryptocurrencies, exemplified by Bitcoin and Ethereum, has emerged as a defining financial phenomenon of the past decade. Within this expanding ecosystem, stablecoins—digital assets engineered to maintain a 1:1 peg with fiat currencies—have ascended as a pivotal nexus linking the crypto sphere with traditional finance, marked by substantial growth in both market capitalization and cross-market liquidity. As digital assets become increasingly integrated into mainstream investment portfolios, the nature of their interconnectedness and the mechanisms of risk transmission with traditional financial markets have attracted significant academic and regulatory attention. This scrutiny has intensified in the post-pandemic era, an environment characterized by heightened global macroeconomic volatility, frequent monetary policy shifts, and recurring geopolitical tensions—factors that have introduced additional layers of complexity to financial risk contagion pathways. These developments raise critical questions: Are the risk spillover effects between digital and traditional financial markets statistically significant? Does the interdependence between these markets exhibit temporal variation? What structural alterations do extreme risk events trigger in their relationship? Addressing these inquiries is essential not only for informing investor asset allocation decisions but also for developing robust frameworks to mitigate systemic financial risks and shape evidence-based regulatory policy.

Early scholarship predominantly examined the asset-like properties of non-stable cryptocurrencies, particularly Bitcoin. Seminal work drew an analogy between Bitcoin and “digital gold,” proposing its potential function as a safe haven during periods of traditional market distress (Baur & Lucey, 2010) [1]. In contrast, Dyhrberg (2016) identified hybrid characteristics, noting that Bitcoin’s price dynamics reflect a blend of market sentiment and macroeconomic factors, exhibiting attributes of both equities and commodities [2]. As the stablecoin market matured, research attention progressively shifted. Empirical evidence suggested that USDT trading volume can serve as a leading indicator for cross-market capital movements (Bouri et al., 2017) [3]. Stablecoins, functioning as a “bridge” between conventional finance and the crypto ecosystem, may themselves introduce novel channels for systemic risk propagation, as they are not immune to speculative attacks, mismanagement, or insufficient collateralization, rendering them a potential source of downside risk rather than a true safe haven (Díaz et al., 2023) [4]. In clarifying the conceptual boundaries, Mulenga et al. (2025) emphasize that stablecoins, as a distinct subset of digital money, deserve particular scrutiny in terms of their payment functions, liquidity management, and the unique governance and regulatory risks they introduce [5]. Regarding market volatility, Bouri et al. (2018) examined the asymmetric volatility spillovers between Bitcoin and other assets during bear and bull markets, finding that Bitcoin primarily acts as a receiver rather than a transmitter of volatility during certain market conditions [6]. Subsequent studies emphasized the central role of macroeconomic policies, regulatory announcements, and social media sentiment in driving price fluctuations (Corbet et al., 2020) [7]. The large-scale entry of institutional investors after 2021 has further reshaped the volatility structure of digital asset markets.

Academic perspectives on the digital-traditional finance nexus have evolved from an initial paradigm of “segmentation” toward one of “integration” (Aldasoro et al., 2025) [8]. Foundational studies largely posited minimal cross-market connection (Basu, 2025) [9]. However, research conducted since the onset of the COVID-19 pandemic has documented a systematic strengthening in these linkages (Conlon et al., 2020; Ji et al., 2020) [10,11]. Analyses employing spillover index models demonstrate that monetary policy signals from the U.S. Federal Reserve can simultaneously impact both equity and digital currency markets. In the context of extreme events, the existing literature confirms that crisis episodes typically amplify cross-market correlations—a phenomenon indicative of “financial contagion”. Nevertheless, much of this research concentrates on isolated shock events, offering limited comparative insight into the heterogeneous impacts arising from different types of extreme events.

Despite valuable contributions across multiple dimensions, three principal gaps remain in the literature. First, regarding variable selection, a predominant focus on non-stable cryptocurrencies like Bitcoin has led to the relative neglect of stablecoins’ core function as cross-market liquidity conduits. This oversight impedes precise identification of the underlying capital flow and risk transmission mechanisms between the two market classes. Second, methodologically, many studies emphasize static correlations during tranquil periods, offering insufficient exploration of dynamic linkage features and the structural transformations induced by extreme events. Consequently, the time-varying properties and state-dependent nature of these interrelationships remain inadequately characterized. Third, with respect to temporal scope, most published studies utilize data ending prior to 2023, thus excluding the pivotal 2023–2025 period characterized by major macroeconomic policy pivots and significant advancements in crypto-asset regulatory frameworks. This limitation constrains the contemporaneity and generalizability of their findings.

To address these gaps, this paper adopts stablecoins as its analytical focal point to investigate the risk spillover effects between digital and traditional financial markets. This approach serves three contributions: (1) shifting the analytical focus from non-stable cryptocurrencies to stablecoin trading activities; (2) systematically examining both static correlations and dynamic co-movements while explicitly distinguishing between normal and extreme-event periods; and (3) extending the timeframe through 2023–2025 to incorporate recent macroeconomic policy shifts and regulatory developments. This integrated framework not only illuminates ongoing structural transformations within the broader financial system but also provides critical insights for safeguarding against systemic instability and offers novel empirical evidence supporting theories of financial market interconnectedness.

### 1.2. Research Objectives and Hypotheses

This paper takes stablecoins as the core entry point of the digital currency market, with specific research objectives including:

It reveals the static correlation characteristics of two types of markets under normal conditions, quantifies the correlation between digital currencies and traditional assets, and judges whether digital currencies have decentralized investment value. It identifies the direction and intensity of cross-market dynamic risk spillovers, tests whether there is an asymmetric transmission relationship between digital currency and traditional financial markets, and roughly determines which party holds the dominant position in risk transmission. It examines the impact effects of extreme risk events on the linkage structure, compares the correlation coefficients between normal and extreme periods, and analyzes the heterogeneous impacts of different types of extreme events (monetary policy, financial crisis, geopolitical conflicts). It characterizes the time-varying characteristics and state dependencies of linkage relationships, and reveals the evolution patterns and key driving factors of the association structure through dynamic correlation analysis of the rolling window. It provides empirical evidence for financial regulation and investment practice, and based on research conclusions, proposes policy recommendations for cross-market risk prevention and asset allocation optimization.

Based on asset pricing theory, risk spillover theory, and extreme risk transmission theory, and in combination with the research gaps in existing literature, this paper proposes the following research hypotheses:

**H1.**  *Under normal conditions, the correlation between the cryptocurrency market and traditional financial markets is weak.*

The correlation coefficients between stablecoin trading volumes and stock, bond, and gold returns are relatively low in absolute value, indicating that the cryptocurrency market is relatively independent and has certain diversification investment value. Corroborating this, Esparcia et al. (2024) examine the diversification benefits of adding cryptocurrencies to equity-based portfolios, further supports this perspective by demonstrating that all types of cryptocurrencies analyzed—including stablecoins—provide diversification opportunities, with stablecoins exhibiting scarce or even negative correlations with equities [12]. Conversely, traditional financial markets internally exhibit stable and significant linkage mechanisms.

**H2.**  *There exist asymmetric weak bidirectional risk spillovers between the cryptocurrency market and traditional financial markets.*

The impact of traditional financial markets (especially the stock market) on the cryptocurrency market is more significant, with cryptocurrency market volatility primarily driven by endogenous factors, and its risk spillover capability to traditional financial markets is limited, overall showing an “asymmetric pattern of traditional market dominance and cryptocurrency market reception.”

**H3.**  *Extreme risk events can structurally reshape cross-market linkage relationships.*

During periods of extreme volatility, the strength or even direction of the correlation between cryptocurrencies and traditional assets can change significantly, and the impact of different types of extreme events is heterogeneous—monetary policy events have the strongest overall impact, financial crises affect the widest range, and geopolitical conflict events have more structural effects.

**H4.**  *The linkage relationship between cryptocurrencies and traditional assets is highly time-varying and state-dependent.*

Cross-market correlation coefficients fluctuate significantly over time and frequently switch direction. Extreme risk events are key catalysts driving structural changes in correlations, and market linkage patterns transform in response to changes in macro-financial conditions.

## 2. Theoretical Foundations and Quantitative Specification of Empirical Models

### 2.1. Asset Pricing Theory

Asset pricing theory provides the foundational framework for analyzing interconnectedness in financial markets. Traditional models, such as the Capital Asset Pricing Model (CAPM), posit that asset prices are determined by factors including the risk-free rate and systematic risk premiums, with capital flows across markets driving prices toward equilibrium (Ciner et al., 2013) [13]. As an emerging asset class, digital currencies exhibit a pricing mechanism that incorporates both conventional characteristics and distinct peculiarities (Yermack, 2024) [14]. On one hand, their prices are influenced by traditional factors such as market supply and demand dynamics and investor risk appetite. On the other hand, due to features like decentralization and anonymity, they are also susceptible to unique drivers including technological advancements, regulatory policies, and prevailing market sentiment.

The pricing mechanism of stablecoins is even more distinctive. Their 1:1 peg to the US dollar results in minimal price volatility, making changes in trading volume the core indicator reflecting the propensity for capital flows (Castrén & Russo, 2026) [15]. When yields in traditional financial markets rise, capital may flow from digital currency markets into traditional ones, leading to a decline in stablecoin trading volume. Conversely, when risk escalates in traditional markets, capital may flood into digital currency markets seeking a safe haven, pushing stablecoin trading volume higher (Bouri et al., 2020) [16]. This process triggers co-movement and risk transmission between the two market types.

### 2.2. Information Asymmetry and Risk Spillover Theory

Drawing on the core tenets of information economics, this study posits that information asymmetry constitutes the microfoundation of risk contagion across financial markets (Galai & Masulis, 1976) [17]. The mechanism operates through three interrelated channels. First, adverse selection distorts pricing: informed traders profit from private knowledge, while uninformed participants price at average quality, undervaluing high-quality assets and overvaluing low-quality ones (Daley & Green, 2012) [18]. When exogenous shocks occur, uninformed investors cannot discern true quality and indiscriminately discount all risky assets, sparking cross-market price spillovers. Second, signaling failures spur herding—credible public signals are scarce, especially in digital currency markets, prompting uninformed investors to mimic observed actions, generating self-reinforcing herding that transmits local liquidity shocks (Campajola et al., 2020) [19]. Third, opacity induces precautionary runs: uncertain about counterparty risk, participants hoard safe assets and freeze short-term funding (Paolo et al., 2024) [20]. Moreover, traditional institutions face high information costs, leading to delayed and exaggerated responses that amplify volatility spillovers and induce nonlinear structural shifts during crises. Overall, information asymmetry impairs price discovery and cross-market information transmission, allowing local disturbances to evolve into systemic contagion (Pasquariello, 2007) [21]. Hence, information asymmetry is argued in the theoretical literature to be a key mechanism for contagion: without informational frictions, rational expectations would dampen transmission; with them, herding, liquidity spirals, and panic expectations forge the contagion chain.

Risk spillover theory posits that risks are transmitted across financial markets through multiple channels, primarily including the information transmission channel, the capital flow channel, and the sentiment contagion channel (Kristoufek, 2015) [22]. The information channel refers to the process where price change information in one market influences investor expectations in another, subsequently triggering price adjustments. The capital flow channel involves investors rebalancing their portfolios across different markets, leading to cross-market fund movements and consequent price co-movements (Smales, 2019) [23]. The sentiment contagion channel describes the diffusion of investor risk preferences or panic sentiment across markets, resulting in the mutual propagation of market volatility.

Risk spillover between digital currency and traditional financial markets operates through these same channels (Chen & Zhang, 2024) [24]. First, information transmission, which means policy changes or economic data releases in traditional markets affect the expectations of investors in digital currency markets. Second, capital flows, which means the existence of stablecoins provides a convenient conduit for cross-market capital movement; the transfer of funds between the two market types directly induces interconnected movements in trading volume and prices (Shu et al., 2025) [25]. Third, sentiment contagion, which means market sentiment reflected by indicators like the VIX fear index simultaneously influences investor behavior in both markets, leading to volatility contagion (Xiao, 2024) [26].

### 2.3. Extreme Risk Transmission Theory

Extreme risk transmission theory posits that extreme events disrupt the normal functioning mechanisms of financial markets, thereby triggering rapid cross-market risk contagion (Gong & Wang, 2025) [27]. Such events primarily affect market linkages through the following channels: First, liquidity shocks—extreme events may precipitate market-wide liquidity crunches, prompting investors to sell various assets to meet liquidity needs, which can lead to synchronized price declines across different markets. Second, sudden shifts in risk appetite—extreme events often induce a sharp decline in investor risk tolerance, sparking panic selling and exacerbating market volatility and co-movement. Third, expectation restructuring—extreme events reshape investor expectations regarding market conditions, altering the underlying logic of asset pricing and consequently transforming inter-market correlations.

Owing to its highly speculative nature and comparatively weak regulatory oversight, the digital currency market exhibits heightened sensitivity to extreme events. In the wake of shocks such as major shifts in macroeconomic policy, financial crises, or geopolitical conflicts, the linkage structure between digital and traditional financial markets may undergo fundamental transformation, with risk spillover effects intensifying considerably (Belke & Beretta, 2020) [28].

### 2.4. Static Correlation Analysis Model

The static correlation analysis model primarily employs the Pearson correlation coefficient to measure the degree of linear correlation between two continuous variables. Here, $\rho_{ij}$ denotes the correlation coefficient between *i* and *j*, $Cov\left(R_{i},R_{j}\right)$ represents their covariance, and Var($R_{i}$) and Var($R_{j})$ are the variances of *i* and *j*, respectively. The formula is as follows:

$$\rho_{ij}=\frac{Cov(R_{i},R_{j})}{\sqrt{Var(R_{i})}\sqrt{Var(R_{j})}}$$

The correlation coefficient ranges from [−1, 1]. The closer its absolute value is to 1, the stronger the linear relationship; the closer it is to 0, the weaker the linear relationship.

After calculating the correlation coefficient, a significance test is required to determine whether the observed correlation is genuine or a result of sampling error. The statistical significance of the correlation is verified using a *t*-test. The test statistic formula is:

$$t=\rho_{ij}\sqrt{\frac{n-2}{1-{\rho_{ij}}^{2}}}$$

where *n* is the sample size. This statistic follows a *t*-distribution with *n* − 2 degrees of freedom. Significance is judged by comparing the calculated value against critical values.

While linear correlation and VAR-based impulse responses provide a transparent benchmark for spillover analysis, recent studies have explored AI and machine learning models for cross-asset risk assessment (Berger, 2023) [29]. These approaches offer potential advantages in capturing nonlinear patterns and high-dimensional interactions beyond pairwise linear correlations. However, they do not automatically resolve identification or data quality issues, and their black-box nature often complicates economic interpretation. The present study therefore retains the VAR framework as the primary methodology, while acknowledging that future research may benchmark its predictive performance against nonlinear alternatives.

### 2.5. Dynamic Linkage Analysis Models

#### 2.5.1. VAR Model Specification

A vector autoregression (VAR) model is constructed to capture dynamic interactions among variables. The model takes the form:

$$Y_{t}=\emptyset_{0}+\emptyset_{1}Y_{t-1}+\emptyset_{2}Y_{t-2}+\cdots +\emptyset_{p}Y_{t-p}+\varepsilon_{t}$$

where $Y_{t}={\left[volumet,sp500t,bondt,goldt,vixt,indext\right]}^{T}$ is a 6-dimensional vector of endogenous variables; $\emptyset_{0}$ is a vector of constants; $\emptyset_{1}$, …, $\emptyset_{P}$ are matrices of coefficients to be estimated; *p* is the optimal lag order (determined by AIC and BIC criteria); and $\varepsilon_{t}$ is a 6-dimensional vector of random disturbance terms, satisfying *E*($\varepsilon_{t}$) = 0 and *E*($\varepsilon_{t}{\varepsilon_{t}}^{T}$) = *Σ* (where *Σ* is the covariance matrix).

The core significance of the model lies in reflecting dynamic influences among variables through lagged terms. The coefficient matrices $\emptyset_{I}$ reveal the strength of mutual transmission of fluctuations across different markets. The goodness-of-fit $R^{2}$ measures the model’s explanatory power for variable fluctuations.

#### 2.5.2. Impulse Response Function (IRF)

The impulse response functions is primarily used to analyze the dynamic impact of a one standard deviation shock to one variable on other variables. It is expressed as:

$$IRF(s)=\frac{\partial Y_{t+s}}{\partial \varepsilon_{t}}$$

where *s* is the lag order (*s* = 0, 1, 2, ……), and $\varepsilon_{t}$ is the standardized disturbance term. By tracing the trajectory of *IRF*(*s*) over *s*, the direction, strength, and persistence of the shock transmission can be clearly presented. The statistical significance of the impulse response is tested using confidence intervals. If the confidence interval does not contain zero, the shock impact is considered significant.

### 2.6. Extreme Period Identification Model

The extreme period identification model primarily uses the *3σ* criterion to identify periods of extreme volatility. The specific formula is:

$$\left|R_{t}-\mu_{R}\right|>3\sigma_{R}$$

where $\mu_{R}$ is the mean of the return series, and $\sigma_{R}$ is its standard deviation. Periods satisfying this condition are defined as extreme volatility periods; the remainder are normal periods. By comparing correlation differences between these two types of periods, the structural impact of extreme events on market linkages is revealed.

### 2.7. Robust Sub-Sample Test Model

The robust sub-sample test model divides the full sample into two sub-samples of equal size (early and late). Correlation coefficients and VAR model coefficients among variables are calculated separately for each sub-sample. The relevant formulas are:

$$∆\rho_{ij}=\left|\rho_{ij,1}-\rho_{ij,2}\right|$$

$$∆\emptyset =\left\|\emptyset_{1}-\emptyset_{2}\right\|$$

where $\rho_{ij,1}$ and $\rho_{ij,2}$ are the correlation coefficients for the first and second sub-samples, respectively; $\emptyset_{1}$ and $\emptyset_{2}$ are the VAR model coefficient matrices for the two sub-samples. $∆\rho_{ij}$ and ∆∅ represent the coefficient differences. If the differences are small and the correlation directions remain consistent, the conclusions are considered robust.

## 3. Data Sources and Preprocessing

### 3.1. Data Selection and Sources

This study investigates the risk spillover effects between digital currencies and traditional financial markets, utilizing daily data from 4 January 2021 to 30 September 2025, as the research sample. This period marks a phase where the digital currency market has progressively entered a stage of scaled development, characterized by a significant increase in the function of stablecoins as a medium for cross-market capital flows (Lu et al., 2025) [30]. Concurrently, this timeframe encompasses a complete macroeconomic cycle, spanning post-pandemic recovery, monetary policy tightening, and subsequent shifts in policy expectations. It also captures a critical phase in the institutionalization of crypto assets and the gradual establishment of regulatory frameworks. Selecting this period facilitates a more precise identification of the time-varying characteristics and structural evolution of risk spillovers.

For the digital currency market, daily trading prices and trading volumes of the two dominant stablecoins, USDT and USDC, which collectively account for over 85% of market capitalization, were selected. For traditional financial markets, daily data series were sourced, including the LBMA gold fix price, the yield on 10-year US Treasury bonds, the closing price of the S&P 500 index, and the closing price of the US Dollar Index. All data were obtained from reputable platforms such as Investing.com, the Wind Database, and Sina Finance, ensuring the authenticity and reliability of the dataset.

### 3.2. Variable Selection and Rationale

The variable system in this study comprises digital currency market variables, traditional financial market variables, and control variables, whose weights are determined via the entropy weight method.

#### 3.2.1. Digital Currency Market Variables

The daily trading prices and trading volumes of USDT and USDC are employed as the primary variables representing the digital currency market. As stablecoins are pegged 1:1 to the US dollar, they serve as the core capital channel bridging the two markets. Their trading volume directly reflects the scale of cross-market capital flows, acting as a key vector for observing risk transmission. Furthermore, USDT and USDC, respectively, cater to retail and institutional liquidity provision, exhibiting strong complementarity that allows for a relatively comprehensive representation of the overall operational state of the stablecoin market (Sukhyi et al., 2024) [31]. Given the relatively low price volatility of stablecoins, changes in their trading volume can more purely reflect the underlying propensity for capital movement, mitigating interference from price fluctuations inherent to other crypto assets.

#### 3.2.2. Traditional Financial Market Variables

Three categories of financial assets, covering distinct risk attributes, are selected as core variables for traditional financial markets: the LBMA gold fix price, the yield on 10-year US Treasury bonds, and the S&P 500 index. Gold, as a traditional safe-haven asset, serves as a key proxy for risk appetite in financial markets; its price movements can reflect the market’s demand for hedging against systemic risk. The S&P 500 index, a benchmark for global equity markets encompassing 500 leading US companies, transmits risk levels prevalent in global stock markets through its fluctuations. The yield on 10-year US Treasury bonds represents the benchmark for the global risk-free rate, directly linked to the pricing logic of fixed-income markets; its variations capture the characteristics of risk transmission within the bond market.

#### 3.2.3. Control Variables

This paper further incorporates the VIX volatility index and the US Dollar Index as control variables. The VIX index directly reflects market expectations for S&P 500 volatility over the next 30 days and is a core indicator of global financial market sentiment and risk aversion. Controlling for VIX helps isolate common fluctuations driven by overall market sentiment, thereby enabling a clearer identification of the unique risk transmission channels between digital currencies and traditional assets. The US Dollar Index serves as a benchmark for global liquidity pricing; its fluctuations directly impact cross-border capital flows and the US dollar-denominated performance of various assets. Including the US Dollar Index in the model helps to exclude distortions in cross-market capital flows caused by exchange rate movements, ensuring that any identified risk spillover effects stem from intrinsic inter-market linkages rather than exogenous influences arising from changes in the US dollar’s value.

### 3.3. Empirical Variable Definition and Processing

To ensure sufficient overlapping periods for analysis across all series and to eliminate dimensional effects while focusing on price variations, all selected data samples are transformed into logarithmic return series using the formula: *R_t_* = ln(*P_t_*/*P_t_*_−1_). Missing values are addressed using linear interpolation. The specific variable definitions and processing are detailed in Table 1 below.

## 4. Empirical Results Analysis

### 4.1. Descriptive Statistics and Stationarity Tests

Table 2 presents the descriptive statistics and stationarity test results for all variables. The mean values indicate that the digital currency trading volume return series exhibits the highest average return at 0.001441, followed by the bond yield return at 0.001246, while the US Dollar Index return displays the lowest mean at 0.000071. This suggests that the average return level in the digital currency market surpasses that of traditional financial markets, providing preliminary evidence for the high-yield characteristic of digital currencies as emerging alternative assets, consistent with the theoretical expectation that nascent assets often generate excess returns during their initial development phases.

Regarding volatility, as measured by standard deviation, the digital currency trading volume return series demonstrates the highest value at 0.318469, substantially exceeding all traditional financial market variables. This is followed by the VIX return series at 0.077191, while the US Dollar Index return exhibits the lowest standard deviation at 0.004523. These findings confirm that volatility in the digital currency market significantly exceeds that of traditional financial markets, highlighting its high-risk profile and aligning with the consensus in existing literature. The VIX index, as a key barometer of market uncertainty, displays a volatility level of 0.077, second only to digital currencies, consistent with its core positioning as a fear gauge, where fluctuations in market sentiment typically exceed those of conventional asset prices.

The skewness statistics reveal asymmetric distribution characteristics across return series. The digital currency trading volume return, S&P 500 return, bond yield return, and VIX return all exhibit positive skewness at 0.433685, 0.012684, 0.111198, and 1.095369, respectively, indicating right-skewed distributions with potential for extreme positive returns. Conversely, gold returns and US Dollar Index returns display negative skewness at −0.359984 and −0.313841, respectively, suggesting left-skewed distributions with potential for extreme negative returns.

Kurtosis values reflect the steepness of distribution tails and extreme value clustering. Both S&P 500 returns and VIX returns exhibit kurtosis exceeding 3 at 6.384117 and 7.770261, respectively, indicating leptokurtic distributions with higher probabilities of extreme returns compared to normal distributions. Digital currency trading volume returns show kurtosis of 3.328669, marginally above 3 and approximating normal distribution. Bond returns, gold returns, and US Dollar Index returns all display kurtosis below 3 at 1.906229, 1.575503, and 1.508608, respectively, indicating platykurtic distributions with lower probabilities of extreme returns and relatively smoother price fluctuations.

The Jarque–Bera normality test results corroborate these distributional characteristics. All return series display Jarque–Bera test statistics at elevated levels with corresponding *p*-values substantially below the 5 percent significance level, leading to rejection of the null hypothesis of normal distribution. This finding aligns with the prevalent non-normality characteristic of financial time series data.

Augmented Dickey–Fuller unit root tests confirm the stationarity properties of all return series. The test statistics for all variables fall well below the critical values at the 5 percent significance level, with *p*-values uniformly at 0.0100. Consequently, the null hypothesis of a unit root is rejected for all series, confirming stationarity at the 5 percent level and enabling subsequent time series analysis without further transformation.

### 4.2. Static Correlation Analysis Between Digital Currency and Traditional Financial Markets

To elucidate the correlation characteristics between digital currency and traditional financial markets, this study examines Pearson correlation coefficients among asset return series, with statistical significance verified through *t*-tests. The results are presented in Table 3.

Regarding correlations between digital currency and traditional assets, volume exhibits a weak negative correlation with the S&P 500 at −0.075, with partial statistical significance. Similarly, volume displays negative correlations with both bond yields and gold returns, though the absolute coefficient values are even smaller. A modest positive correlation of 0.130 emerges between volume and the VIX index, potentially suggesting that digital currency trading activity intensifies during periods of heightened market volatility and uncertainty. These findings indicate relatively weak correlations between digital currency markets and traditional financial markets, with some evidence of mild counter-cyclical movement patterns. This provides some support for the relative independence of digital currency markets from traditional financial markets, suggesting potential diversification benefits for digital currencies.

Within traditional financial markets, the S&P 500 demonstrates a strong negative correlation with the VIX index at −0.7643, consistent with the conventional market logic of equity declines accompanying volatility increases. Gold exhibits a significant negative correlation with the US Dollar Index at −0.4477, reflecting its dollar-denominated pricing mechanism. Bonds and gold display a notable negative correlation at −0.2802, potentially capturing the interplay between inflation expectations and safe-haven demand. The S&P 500 shows a negative correlation with the US Dollar Index at −0.2480, aligning with the suppression effect of a strong dollar on multinational corporate earnings. All these significant correlations pass tests at the 1 percent level, indicating stable internal linkages within traditional financial markets.

Statistical significance testing of correlations reinforces these findings. The t-statistics for the volume-S&P 500 relationship and the volume-VIX relationship are −2.60 and 4.52, respectively, both significant at the 1 percent level, confirming that these correlations possess statistical meaning rather than arising from random fluctuations. Conversely, the t-statistics for correlations between volume and bond yields, gold returns, and the US Dollar Index exhibit relatively small absolute values without statistical significance, suggesting these weak correlations lack statistical reliability and may result from sampling variation.

### 4.3. Dynamic Linkage Analysis

#### 4.3.1. Vector Autoregression Model Specification and Interpretation

(1) Model specification and selection of optimal lag order

While the preceding correlation analysis reveals static association characteristics between digital currency and traditional financial markets, it fails to capture the dynamic evolution of these relationships over time. To better understand the dynamic linkage mechanisms and volatility transmission effects between digital currency and traditional financial markets, this study proceeds to estimate a vector autoregression model. Prior to model estimation, optimal lag length selection is performed using the Akaike Information Criterion and Bayesian Information Criterion. Both criteria identify one lag as optimal, with corresponding AIC value of −11.6405 and BIC value of −11.5547, indicating satisfactory model fit and ensuring estimation reliability.

The VAR model estimation results reveal heterogeneity in equation fit across markets. The S&P 500 return equation demonstrates strong explanatory power with an R^2^ of 0.6012. The VIX equation exhibits moderate explanatory power with an R^2^ of 0.2061. In contrast, the equations for digital currency trading volume and bond yields show relatively low R^2^ values of 0.1050 and 0.0697, respectively, suggesting that volatility in digital currency and bond markets is influenced by own lags and other market lags only to a limited extent, with potentially important drivers not captured by the current model specification. The trace of the residual covariance matrix stands at 0.091, indicating reasonable model stability. The optimal lag length of one period suggests that dynamic cross-market effects operate primarily through short-term transmission channels, with lagged effects most pronounced at the one-period horizon and weaker long-term linkages.

(2) Model System Testing

To ensure the validity of statistical inference in the VAR model, this paper conducts rigorous diagnostic tests on the model residuals, the results are shown in Table 4. First is the system stability test: the maximum modulus of all characteristic roots is far less than 1, indicating that the VAR system satisfies the stationarity condition, the impact of shocks decays over time, and the system has inherent stability. Second is the residual autocorrelation test: the Portmanteau Q test statistic is 0.5488, with a corresponding *p*-value of 1.0000, which cannot reject the null hypothesis of “no autocorrelation in residuals” at the 5% significance level, indicating that the model effectively captures the linear dynamic information of the series. Finally, for the residual heteroscedasticity test, the *p*-values of the ARCH-LM test all correspond to probabilities below 0.05, indicating that the residuals of each equation have significant conditional heteroscedasticity.

Overall, the above results indicate that the VAR model is reliable in estimating regression coefficients. The dynamic influence between traditional markets and digital currency markets is primarily transmitted in the short term (one-period lag), with weak long-term linkage features. The presence of conditional heteroscedasticity in the model residuals also provides factual evidence for subsequent volatility analysis under extreme market conditions.

#### 4.3.2. Granger Causality Test

To accurately identify whether there exists a statistically stable “lead-lag” guiding relationship between the digital currency market and traditional financial markets, this paper conducts a Granger causality test based on the VAR(1) model. The results are presented in Table 5.

As shown in Table 5 at the 5% significance level, none of the cross-Granger causality tests involving “Digital Currency Trading Volume” pass the significance threshold. This indicates that, at a daily frequency, there is no statistically robust linear predictive guiding relationship between the core variable of this study and traditional financial markets. However, this does not negate the “cross-market co-movements” identified earlier; rather, it precisely characterizes their specific manifestations:

(1) Instantaneous Shocks and Rapid Attenuation: The non-significant GC test merely implies that today’s prices cannot serve as reliable signals for forecasting tomorrow’s price fluctuations. This is highly consistent with the VAR impulse response results—cross-market shocks are predominantly concentrated at lags 0 and 1 (contemporaneous instantaneous shocks) and quickly converge to zero after lags 2 and 3. These “short-lived, fast-dissipating” shocks are inherently insufficient to form a stable statistical inertia within the day-to-day progressive lag test framework.

(2) Revealing Efficiency in Asset Pricing and Logical Decoupling: This outcome reflects the “semi-strong form efficiency” characteristics of the market. The capital flows triggered by macro shocks are absorbed extremely quickly within the same day, manifesting as contemporaneous co-movement between stablecoin trading volumes and asset prices (as illustrated by the DCC dynamic correlation diagram). The rapidity of capital absorption prevents cross-market arbitrageurs from establishing sustainable “lag arbitrage rules”. The insignificance of the GC test actually verifies the high efficiency of cross-asset information transmission.

(3) Confirming Weak Exogeneity and Asymmetric Transmission: Combined with the significant disparity in R-squared values from the preceding VAR model (R^2^ = 0.1050 for the digital currency equation, far lower than the R^2^ = 0.6012 for the S&P 500 equation), this result further corroborates the strong endogeneity of digital currency volatility, which is predominantly driven by its own internal narratives and speculative sentiment. Traditional markets struggle to provide a persistent leading guide, and vice versa. This suggests that cross-market structural mutations are more likely to be instantaneous contagions within specific time windows, rather than stable transmission mechanisms under normal conditions.

#### 4.3.3. Variance Decomposition

The variance decomposition results in Table 6 indicate that the fluctuations in digital currency trading volume are predominantly explained by its own shocks. In Period 1, the self-contribution rate reaches 99.535%. Although it declines slightly in subsequent periods, it consistently remains above 80% during the first four periods. This suggests that the volatility in the digital currency market exhibits strong endogeneity, with external markets exerting limited influence on it in the short term.

From the perspective of traditional financial assets, the variance contributions are modest and lack a stable lag structure. The S&P 500 contributes 4.655% in Period 2, bonds reach 14.750% in Period 3, and gold contributes 5.298% in Period 4. However, these magnitudes are small and fluctuate irregularly across horizons. When viewed alongside the non-significant Granger causality results, these figures indicate that any lagged transmission from traditional markets is weak and episodic, rather than reflecting a reliable predictive relationship. The self-contribution of digital currency volatility remains above 80% throughout, confirming its strong endogenous nature.

Overall, the variance decomposition of digital currency trading volume further validates the previous conclusions: the volatility in the digital currency market is primarily self-driven. Traditional financial assets exhibit modest and irregular contributions to stablecoin volume variance, but the overall contributions are limited and unstable. In cross-market risk transmission, the digital currency market predominantly plays the role of a receiver.

#### 4.3.4. Impulse Response Analysis

**Figure 1:**
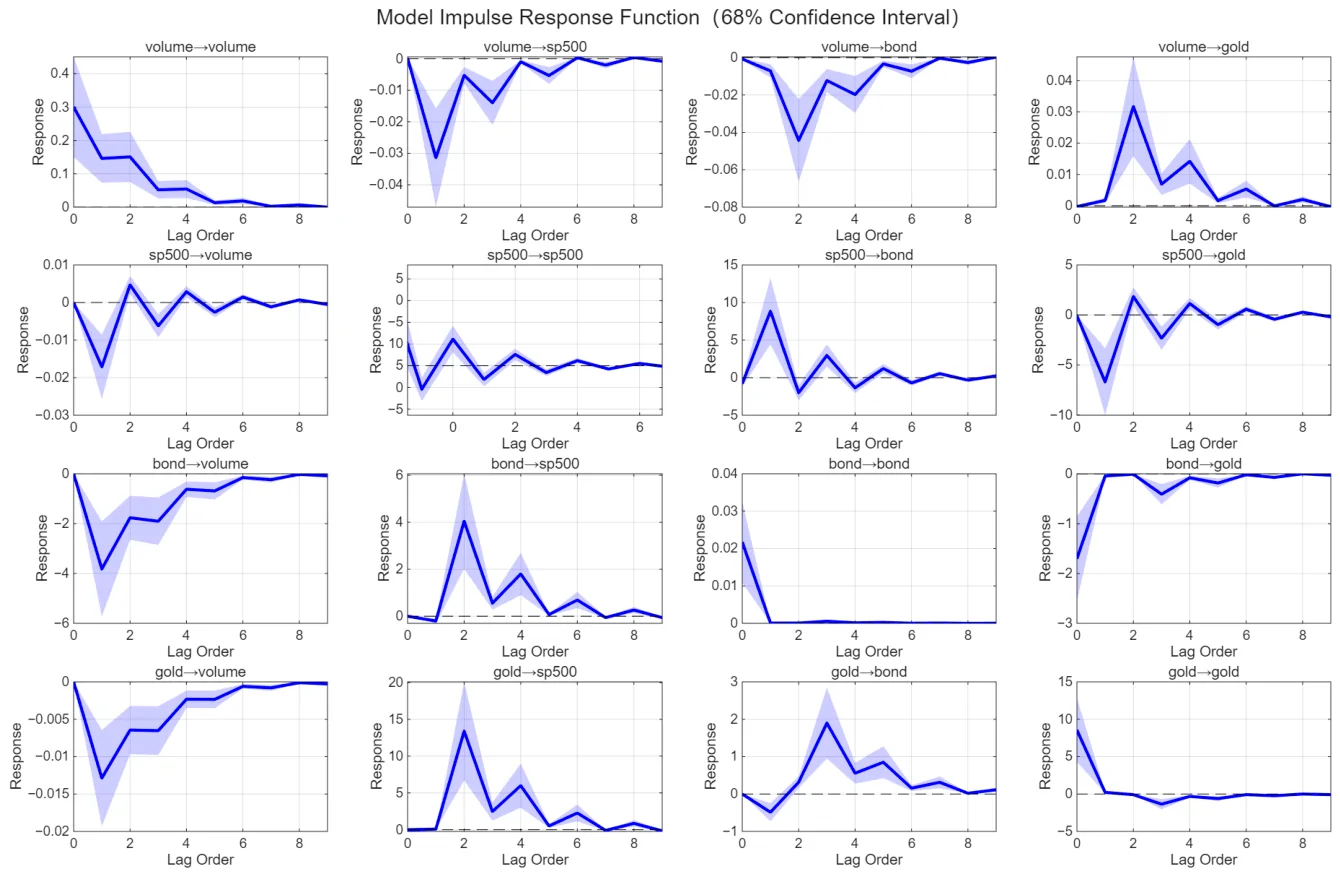
Impulse response functions. Note: The solid blue line denotes the point estimate of impulse-response function. The light-blue shaded area refers to the 68% confidence interval (±1 standard error).

To further investigate the dynamic interactions and risk transmission mechanisms between digital currency and traditional financial markets, this study generates impulse response functions. The results, presented graphically below in Figure 1, clearly illustrate the direction, magnitude, duration, and statistical significance of shocks, providing empirical evidence regarding contemporaneous and lagged market responses.

(1) Heterogeneity in Own-Shock Responses

The response patterns of each market to its own shock directly reflect the endogeneity and persistence of volatility. Digital currency trading volume exhibits the most pronounced response to its own shock, with the contemporaneous impact reaching approximately 0.3 at period zero, followed by rapid monotonic decay and convergence toward zero by period six. This pattern clearly indicates significant volatility clustering in digital currency markets, where current period abnormal activity substantially elevates subsequent volatility in the near term, though the persistence of such effects remains relatively limited, typically dissipating within approximately one week of trading. By contrast, traditional financial markets display different response patterns to own shocks. The impulse response curves for the S&P 500, bond yields, and gold returns show initial magnitudes substantially smaller than those of digital currencies, with peak absolute responses below 0.02, and exhibit more gradual decay paths. This suggests that while volatility persistence exists in traditional assets, the self-reinforcing effect of volatility is comparatively weaker, with potentially faster market adjustment or information absorption.

(2) Asymmetry and Weakness in Cross-Market Risk Spillovers

Regarding spillover effects from digital currency to traditional markets, a positive shock to digital currency trading volume exerts generally negative effects on traditional asset returns. The response of S&P 500 returns remains negative throughout the entire ten-period forecast horizon, with a peak negative response of approximately −0.003 occurring at lag two, followed by gradual decay. Response patterns for bond yields and gold returns resemble those for equities, displaying modest negative effects. However, the magnitudes of these response curves remain extremely small, with peak absolute values below 0.01, and the corresponding 68 percent confidence intervals contain zero for the majority of lag periods. This indicates that while the direction of influence is negative, the negative spillover effects from increased digital currency activity to traditional markets lack statistical robustness and demonstrate limited economic significance. These findings support the observation of some degree of market segmentation under normal conditions.

Concerning reverse spillover effects from traditional markets to digital currency, shocks originating in traditional markets exhibit some asymmetry and complexity in their effects on digital currency trading volume. A positive shock to S&P 500 returns produces a sustained and relatively more pronounced negative effect on digital currency trading volume, with the response curve remaining consistently below zero and confidence intervals excluding zero for certain lag periods. A positive shock to bond yields generates an effect path showing weak negative responses contemporaneously and at lag one, subsequently transitioning to sustained positive responses beginning at lag two, though magnitudes remain extremely small throughout. Meanwhile, a positive shock to gold returns produces weak negative effects on digital currency trading volume.

(3) Transitory Nature of Shock Effects and System Stability

All impulse response functions, whether own-responses or cross-responses, converge toward zero after approximately six to eight periods. This common characteristic indicates that shocks originating in any market, whether internal or external, produce only transitory effects, with the overall system exhibiting dynamic stability such that shocks do not generate persistent divergent effects. This finding aligns with the fundamental logic of efficient market hypotheses, where new information is gradually absorbed and incorporated into prices.

Synthesizing the impulse response analysis reveals asymmetric and weak bidirectional risk transmission between digital currency and traditional assets. Among these transmission channels, traditional equity markets exert relatively more pronounced effects on digital currency, while the high volatility characteristic of digital currency markets produces stronger persistence in own-effects compared to spillover effects on traditional markets. The statistical insignificance characterizing most cross-market effects further suggests that, during the normal periods represented by the sample, the two markets operate with relative segmentation, with strong risk contagion not constituting a pervasive feature.

#### 4.3.5. Extreme Scenario and Tail Risk Event Impact Analysis

To thoroughly investigate the differential impacts of various types of extreme risk events on financial markets, this study examines nine representative extreme event windows, further analyzing the heterogeneity and structural effects of extreme event shocks.

(1) Heterogeneous Impact of Extreme Events on Market Volatility

**Figure 2:**
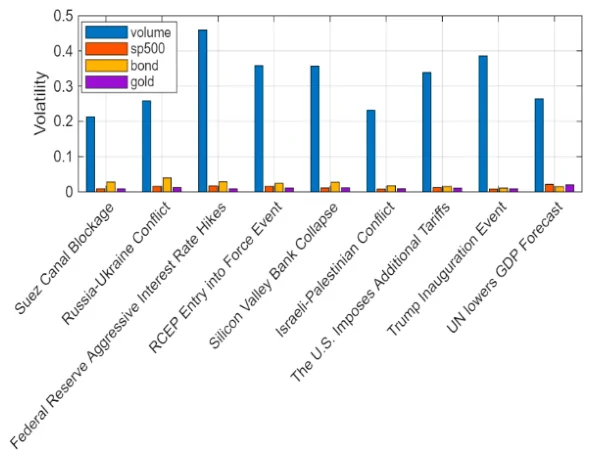
Market volatility during extreme events.

As shown in Figure 2, overall, digital currency markets demonstrate higher sensitivity to various extreme events compared to traditional financial markets, consistent with their inherent high-volatility characteristics. Notably, during the Federal Reserve aggressive rate hike event window, digital currency volatility peaks at 0.4595, substantially exceeding all other events, underscoring the extreme vulnerability of digital assets to macroeconomic policy shocks such as global liquidity tightening and risk-free rate surges. This validates the characterization of digital currency markets as predominantly driven by liquidity and speculative sentiment, exhibiting exceptional sensitivity to marginal changes in interest rate policy.

The Federal Reserve’s aggressive rate hike event generates the strongest volatility impact within the sample period for both the S&P 500 index at 0.0171 and bond markets at 0.0288. This indicates that monetary policy tightening characterized by rapid interest rate increases most directly impacts traditional equity and bond markets as core financing and pricing venues through discount rate elevation and growth concerns. The Russia–Ukraine conflict produces a significant volatility impact in bond markets at 0.0394, while the UN GDP forecast downgrade event most substantially impacts gold markets at 0.0200. These patterns, respectively, reflect the disturbance to fixed income markets from safe-haven demand triggered by geopolitical conflict, and the repricing of traditional ultimate safe-haven assets in response to deteriorating growth prospects. The Silicon Valley Bank collapse event generates pervasive high volatility across equity, bond, and gold markets, illustrating the broad contagion effects of regional banking crises transmitted through liquidity panic and credit risk reassessment channels.

**Figure 3:**
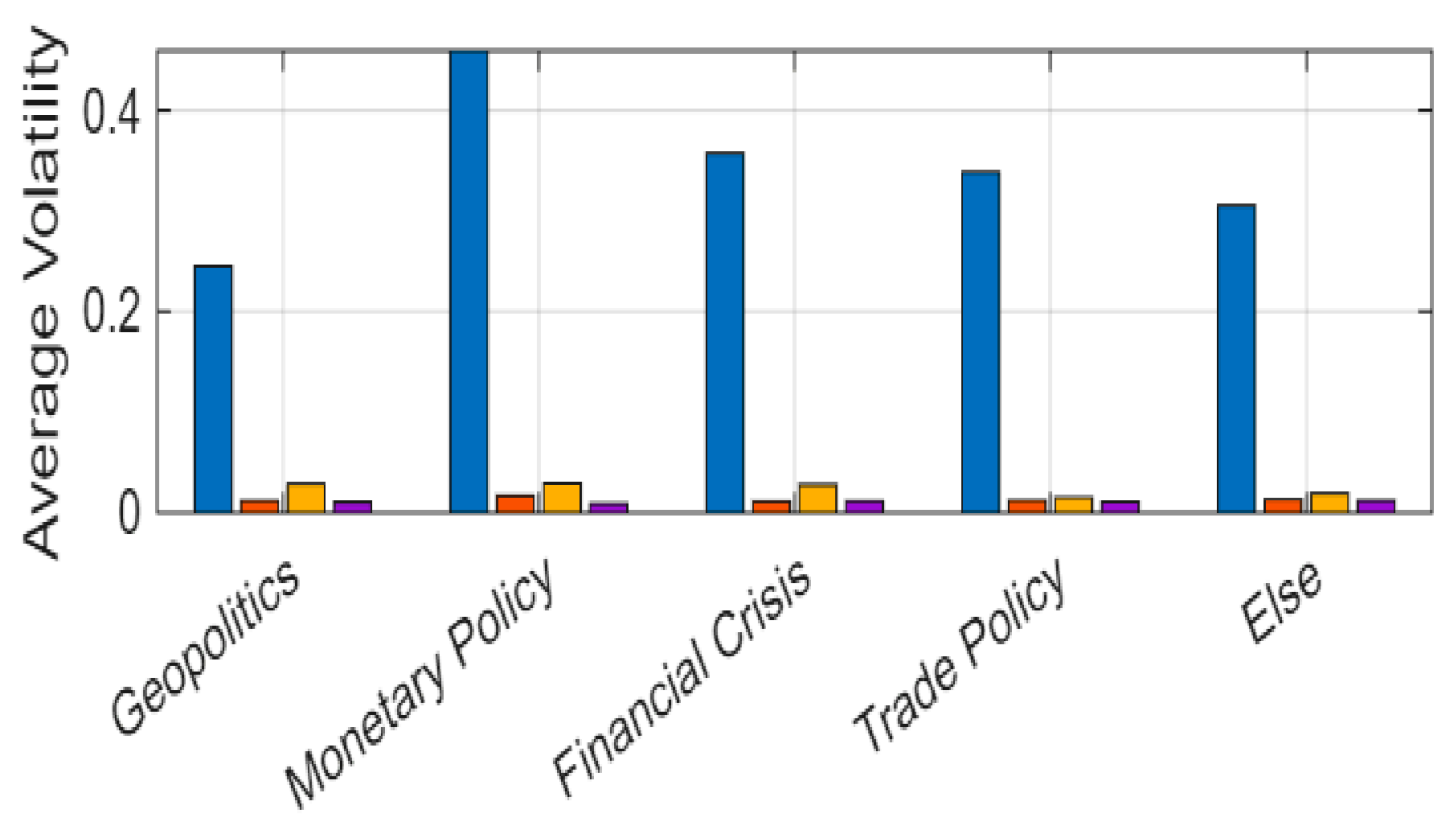
Average impact of different types of events. Note: The colors in the figure represent the markets in the same way as explained in the legend of the Figure 2.

Figure 3 further summarizes the average impact intensity of different types of extreme events on various assets.Considering average volatility impacts comprehensively, macroeconomic policy events exert the highest overall impact intensity on the financial system; financial crisis events follow, with wide-ranging effects; geopolitical conflict events demonstrate relatively lower average impact intensity while exhibiting more structural effects with pronounced impacts on specific safe-haven assets.

(2) Structural Reshaping of Cross-Market Correlations During Extreme Events

**Figure 4:**
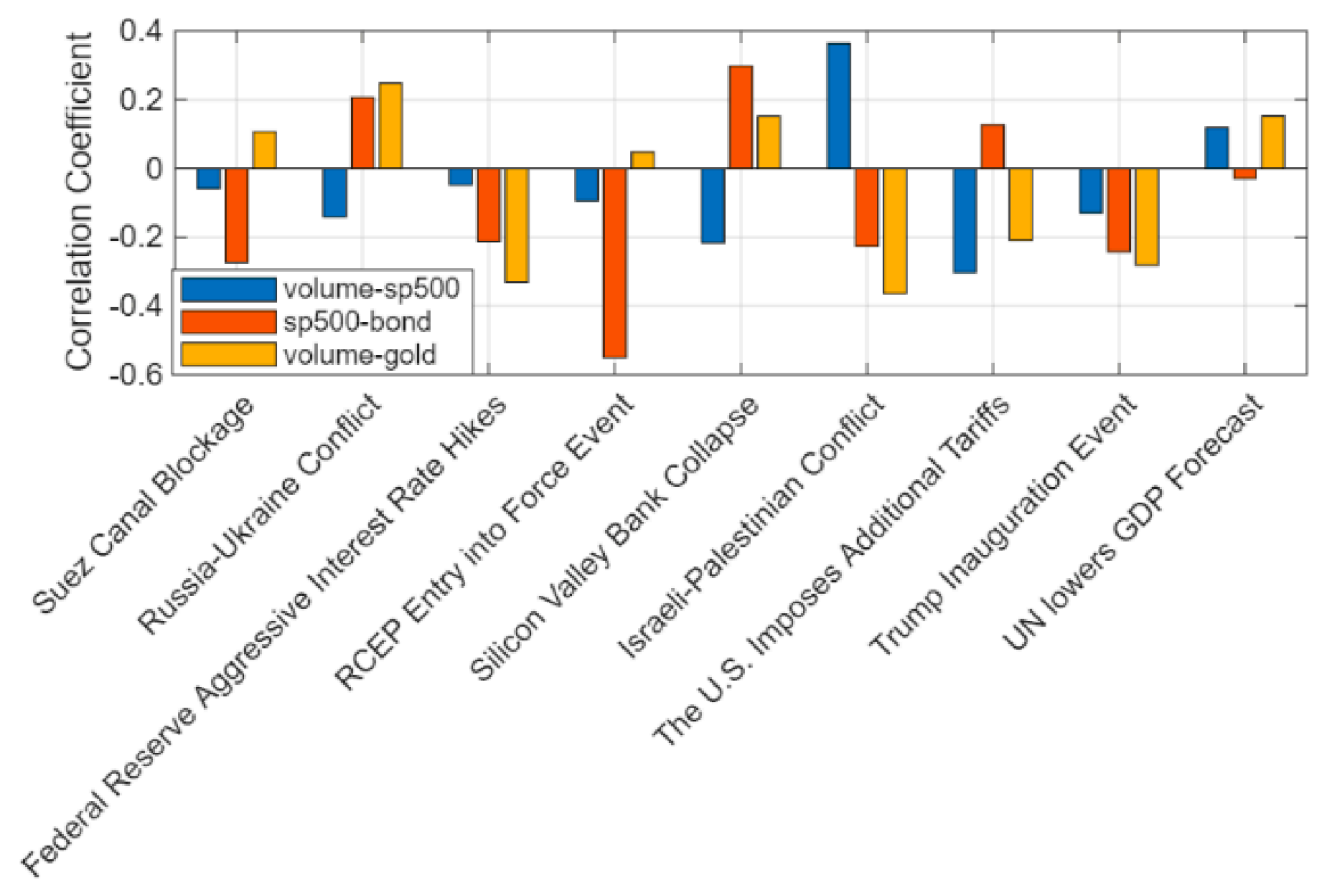
Market correlation during extreme events.

As shown in Figure 4, during the Israel-Palestine conflict, the correlation between digital currency and equities surges to 0.3637, indicating strong risk comovement and suggesting that during deepening geopolitical crises, digital assets may be traded by some market participants as high-beta risk assets similar to equities. Conversely, during the US tariff increase and Silicon Valley Bank collapse events, this correlation declines to −0.3030 and −0.2165, respectively, displaying either safe-haven divergence or differential performance under simultaneous selling pressure. As further quantified in Table 7, the correlation between stablecoin trading volume and bonds reverses from the normal-period weak negative correlation of −0.064 to a positive correlation of 0.2161 under extreme market episodes. This may reflect the dominance of liquidity pursuit effects during crises, where investors simultaneously sell digital currency and bonds to obtain liquidity, causing short-term convergence in price movements and temporarily disrupting traditional safe-haven asset logic.

During the Federal Reserve’s aggressive rate hike period, digital currency and gold exhibit a strong negative correlation at −0.3315, whereas during the Russia–Ukraine conflict, they show a positive correlation at 0.2479. This suggests that when facing interest rate shocks, the pricing logic of digital currency and gold may confront trade-offs between real interest rates and speculative attributes; while facing geopolitical risks, both may simultaneously benefit from safe-haven capital inflows. Extreme events similarly impact long-standing correlation patterns within traditional markets. For instance, during the RCEP implementation event window, the equity-bond correlation plunges to −0.5500, exhibiting extremely strong negative correlation, potentially reflecting extreme effects on relative equity and bond valuations from dramatic fluctuations in growth expectations and inflation expectations under significant trade agreement shocks.

These findings indicate that extreme events not only amplify volatility but can also induce structural changes in cross-market correlations. The nature of extreme risk events determines market participants’ asset allocation logic, consequently influencing cross-market linkages. Macroeconomic policy events primarily affect linkages through funding cost channels, and geopolitical events transmit mainly through market expectation channels. In contrast, financial crisis events trigger cross-market risk comovement through liquidity channels.

#### 4.3.6. State-Dependent Correlation Changes Under Extreme Scenarios: Normal Versus Extreme Period Comparison

**Figure 5:**
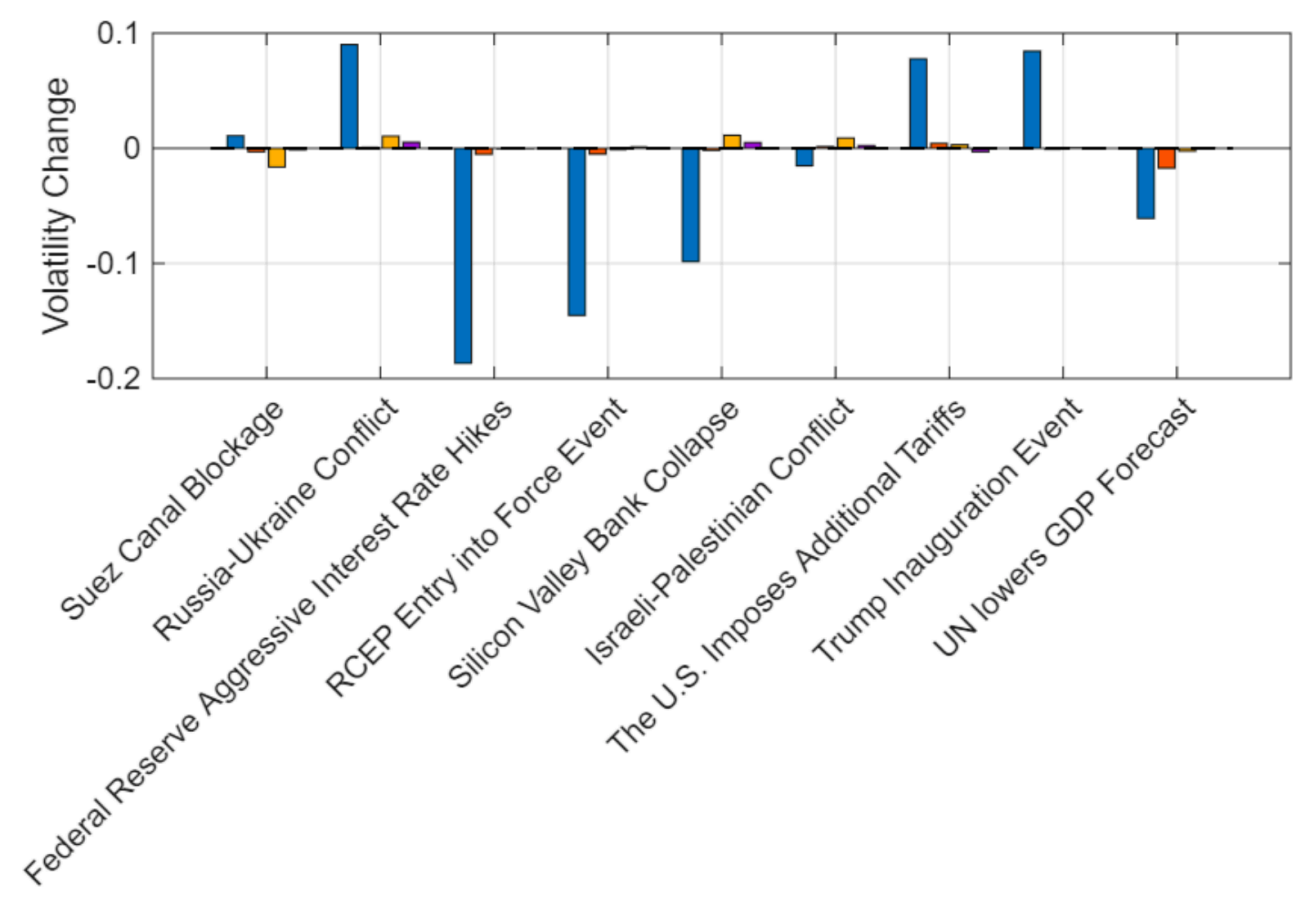
Risk spillover changes before and after the event (volatility difference). Note: The colors in the figure represent the markets in the same way as explained in the legend of the Figure 2.

As shown in Figure 5, to systematically and quantitatively reveal the fundamental effects of extreme market stress on asset correlation structures, this study identifies extreme volatility periods based on the standardized residuals from the VAR model, applying the 3σ criterion. Specifically, for each trading day, if the absolute value of the standardized residual of any variable (trading volume, stocks, bonds, gold, the VIX, and the US dollar index) exceeds 3, that day is marked as an extreme day; consecutive extreme days are then merged into a single extreme interval to avoid double-counting the same shock. Ultimately, 59 extreme intervals are identified from the full sample; trading days falling within these intervals are classified as the extreme period, while the remaining days are classified as the normal period, thereby achieving a strict separation of the two market regimes. To further determine whether the variations in correlation coefficients during these periods are due to systemic structural shifts or short-term random disturbances, this paper introduces a two-sample difference test based on the Fisher Z transformation, presented in Table 7. This comparative analysis not only validates observations from the preceding event study but also statistically confirms how extreme risks systematically reshape market linkages.

(1) Strong Statistical Evidence: Structural Reversal in the Digital Currency-Bond Relationship

From the perspective of rigorous hypothesis testing, the changes in correlation coefficients for the vast majority of asset pairs fail to pass the significance test at the 5% level. However, the relationship between “digital currency and bond yields” exhibits distinctly different statistical characteristics. Its weakly negative correlation during normal periods (−0.0640) reverses into a positive correlation during extreme periods (0.2161), with a difference as high as 0.2801, which is statistically highly significant (*p*= 0.0383). This result statistically confirms the structural reshaping of the co-movement logic between the stablecoin market and traditional bond markets under extreme risk events. Under normal conditions, their distinct positioning as “high-risk speculative assets” and “traditional safe-haven assets” is supplanted during extreme events by the “liquidity runs and synchronized multi-asset sell-offs” effect, fundamentally altering the capital flow logic underlying risk-hedging and repatriation between the two markets.

(2) Dialectical Unity of Numerical Trends and Statistical Robustness: Enhanced Co-movement in Other Asset Pairs

Although the *p*-values indicate that the differences for the remaining asset pairs do not reach the threshold of statistical significance, one cannot simply rule out the trend of enhanced co-movement from an economic perspective, primarily for the following reasons: First, the sample of extreme periods accounts for only a very small proportion of the full sample (59 observations). The limited sample size leads to reduced statistical power, inflated variance, and consequently, larger *p*-values. Second, both the impulse response analysis and rolling-window dynamic charts presented earlier have clearly demonstrated that during extreme periods, the absolute values of correlations among assets generally magnify, and localized directional reversals even occur (e.g., the negative correlation between volume and gold intensifies, while the positive correlation between the S&P 500 and gold strengthens). These numerical morphological changes are highly consistent with prior observations and serve as a direct manifestation of cross-asset risk contagion during shock windows.

Overall, aside from the digitally bond reversal (*p* = 0.0383), the correlations between digital currencies and other assets show systematic amplification in absolute values during extreme periods, though statistical significance is constrained by the limited sample of extreme events (59 observations). This asymmetry highlights that cross-market co-movement is “universally intensified” under extreme risk, but the strength of evidence varies across asset pairs. The digital-bond reversal represents the most robust transmission dimension and a priority indicator for cross-market risk early-warning systems.

#### 4.3.7. Time-Varying Correlation Characteristics: Rolling Window Dynamic Correlations

To examine the impact of different types of shocks—such as geopolitical events, monetary policy changes, and financial crises—on financial markets, this study selects nine representative extreme risk events that occurred between March 2021 and May 2025. These events include the Suez Canal blockage, the Russia–Ukraine conflict, the Fed’s aggressive rate hikes, the implementation of RCEP, the collapse of Silicon Valley Bank, the Israel–Hamas conflict, U.S. tariff hikes, the Trump inauguration, and the UN’s downward revision of global GDP forecasts. For each event, we construct an event window spanning 15 trading days before and 15 trading days after the event date, yielding a uniform window length of 31 trading days. This consistent window design ensures comparability and robustness across all events analyzed. The definition of extreme risk event periods is in Table 8.

**Figure 6:**
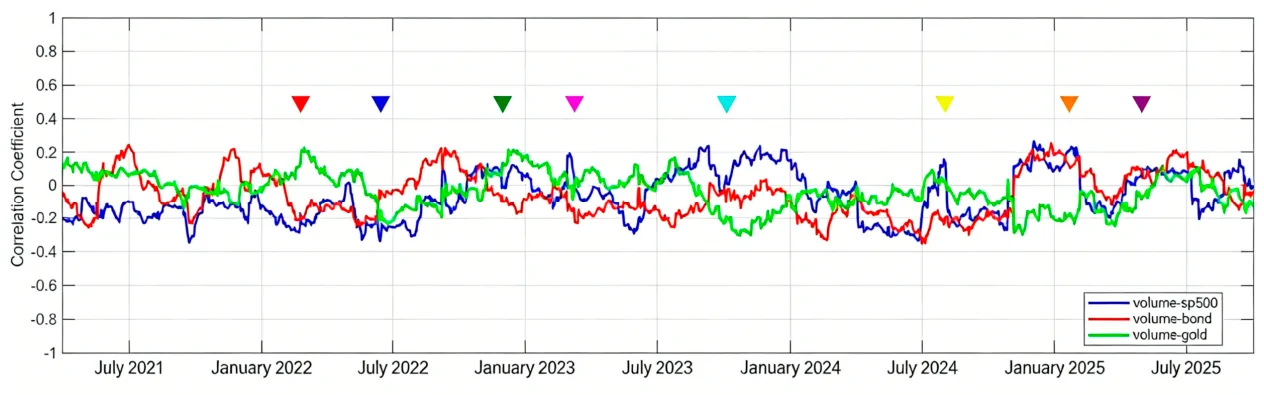
The dynamic correlation between digital currencies and other markets. Note: Different colored triangles represent different extreme risk events.

**Figure 7:**
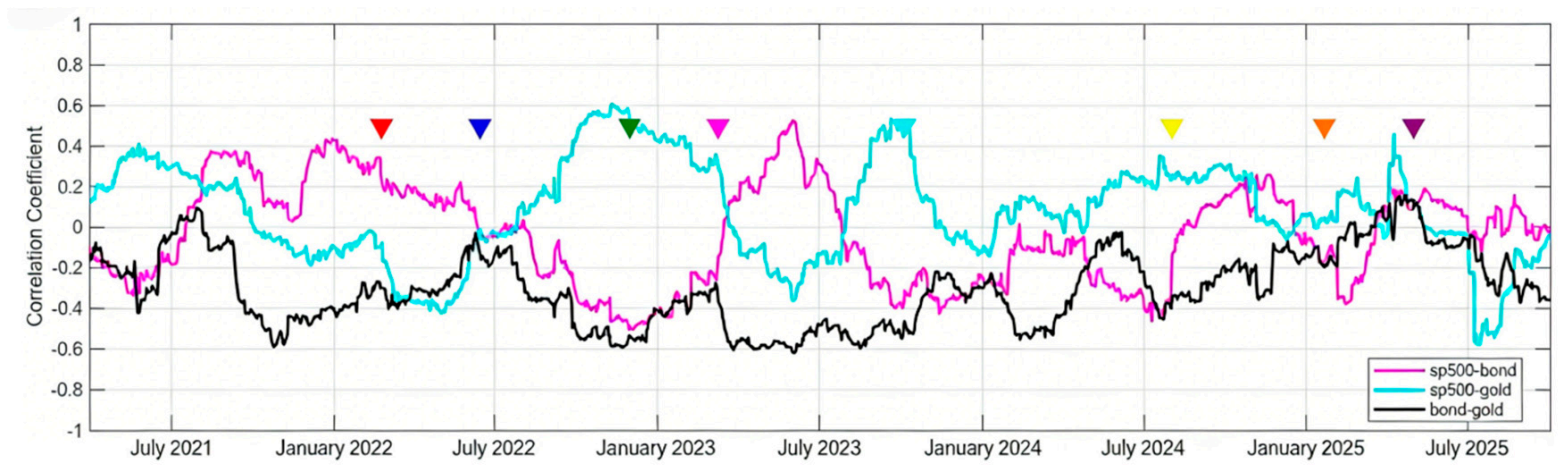
Dynamic correlation between traditional financial markets. Note: Different colored triangles represent different extreme risk events.

Recognizing the limitation of static correlation coefficients in capturing time-varying correlation structures, this study employs rolling window estimation to compute dynamic correlations, further investigating the impact of extreme events on market linkage patterns. The results are presented in Figure 6 and Figure 7.

(1) Fragility and State Dependence of Digital Asset-Traditional Asset Linkages

The upper panel of figures clearly depicts the evolution of dynamic correlations between digital currency trading volume and three major traditional financial assets. Throughout the entire sample period, all three curves oscillate widely around zero, exhibiting no stable long-term trends or mean-reverting tendencies. Examining the digital currency-equity correlation curve, its value range swings dramatically between −0.4 and 0.2: plunging below −0.4 during the Russia–Ukraine conflict escalation in early 2022, followed by a rapid rebound, yet transitioning to sustained positive correlation approaching 0.2 during specific phases from late 2023 to early 2024. Such frequent and substantial directional reversals indicate that digital asset-traditional asset linkages are not governed by any constant economic or financial logic, but instead exhibit high instability and dependence on economic states. The direction and intensity of linkages prove highly susceptible to abrupt changes driven by short-term market sentiment, capital flows, or specific event impacts. These findings, from a dynamic perspective, corroborate and deepen the conclusion of weak linkages from static analysis, suggesting not only low average correlation but also fundamental instability in that low correlation itself.

(2) Disturbance and Regime Switching in Traditional Financial Market Internal Linkages

The lower panel of figures reveals the dynamic characteristics of correlations among mature asset classes including equities, bonds, and gold. All three curves exhibit significant volatility, particularly pronounced during the 2022–2023 period. For example, the equity-bond correlation remains negative for most periods, yet exhibits sharp elevation above zero with brief positive excursions around the March 2023 Silicon Valley Bank crisis. This suggests, to some extent, that even long-established correlation patterns among traditional financial markets may experience temporary disruption or reversal when confronted with extreme liquidity shocks or systemic stress events. During crises, safe-haven demand and liquidity requirements may overwhelm traditional asset allocation logic, inducing synchronous price movements or comovement across different asset classes. This demonstrates that traditional market correlations are not inherently stable but undergo regime switching in response to evolving macro-financial conditions.

(3) Extreme Risk Events as Critical Catalysts for Correlation Breaks

The inflection points marking sharp turning points, steep rises, or declines in most correlation curves exhibit high temporal coincidence or close proximity with the colored triangular markers indicating various extreme risk events. This spatial-temporal correspondence provides compelling visual evidence that external extreme risk event impacts serve as the most critical catalysts driving structural breaks in dynamic correlations both between digital assets and traditional assets and within traditional asset classes. Whether geopolitical crises, abrupt macroeconomic policy shifts, or financial system risk outbreaks, these events rapidly reshape price co-movement patterns across asset classes by altering investor risk preferences, adjusting asset allocation strategies, triggering cross-border capital flows, or inducing liquidity constraints. Consequently, any correlation analysis that neglects major event contexts may severely misjudge actual market linkage risks.

#### 4.3.8. Robustness Checks

To ensure the reliability of empirical conclusions, this study employs two robustness verification methods in Table 9: subsample analysis and correlation stability testing. Subsample analysis divides the full sample into two equal halves, comparing the coefficients of digital currency market effects on traditional financial markets. The coefficient in the first subsample stands at −0.538873, while the second subsample coefficient is −0.025941. Although absolute coefficient values differ, both maintain negative effects, indicating stability in the negative digital currency-equity linkage, with only impact intensity varying over time.

Detailed correlation robustness test results are presented in Table 9. The volume-S&P 500 correlation coefficient evolves from −0.1201 in the early period to −0.0171 in the later period, yielding a change magnitude of 0.1030. The volume-gold correlation transitions from 0.0124 in the early period to −0.0702 in the later period, with a change magnitude of 0.0826. Correlation changes among other variable pairs remain within reasonable ranges. Overall, the direction of correlations across variable pairs does not undergo a permanent reversal at the full-sample average level, confirming the robustness of the baseline long-term linkage patterns. Importantly, this baseline stability does not contradict the transient, state-dependent sign reversals documented during extreme event windows (e.g., the volume–bond reversal in Section 4.3.5); rather, it highlights that such reversals are episodic stress-induced mutations. Together, these findings reinforce the state-dependent nature of cross-market linkages, as posited in Hypothesis 4.

**Figure 8:**
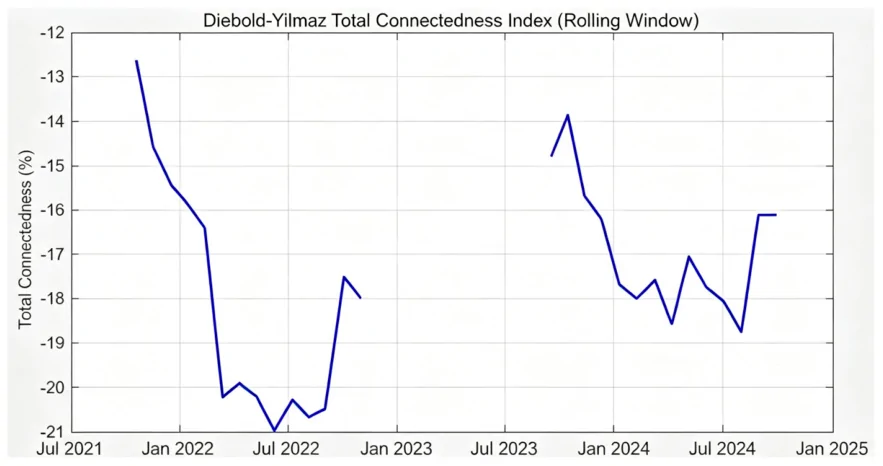
Diebold–Yilmaz total connectedness index (Rolling window).

Furthermore, this study employs the rolling-window Diebold–Yilmaz connectedness index to characterize the time-varying overall risk connectedness level of the system. The results are presented in Figure 8.

In terms of the time-series trend, the total connectedness index of the system consistently remains within the negative range throughout the sample period, oscillating between [−21, −13]. This indicates that the overall system risk connectedness level formed by digital currencies and traditional financial markets is relatively low, and the cross-market risk transmission intensity is limited, which is consistent with the market segmentation conclusions derived from the previously discussed VAR model and variance decomposition.

Simultaneously, the total connectedness index exhibits pronounced time-varying fluctuation characteristics. From the second half of 2021 to mid-2022, the index continued to decline, reaching its lowest point in the sample period around mid-2022, corresponding to the macroeconomic backdrop of aggressive interest rate hikes by the Federal Reserve and the tightening of global liquidity. The index showed a phased rebound in mid-2023, followed by range-bound oscillations throughout 2024. This indicates that cross-market connectedness is not static but adjusts in response to changes in the macroeconomic environment and market sentiment. Overall, however, there is no prolonged or sustained high-connectedness state, with risk spillover primarily exhibiting short-term and episodic characteristics.

By synthesizing the results of the three categories of robustness checks—namely, sub-sample regressions, correlation stability tests, and the Diebold–Yilmaz dynamic connectedness index—the core conclusion of this paper, which posits that “under normal conditions, the co-movement between digital currencies and traditional financial markets is weak, with limited risk spillover effects,” demonstrates strong robustness.

## 5. Conclusions and Policy Recommendations

### 5.1. Conclusions

This study systematically investigates the risk spillover effects and dynamic linkage mechanisms between digital currency and traditional financial markets using daily data from 4 January 2021 to 30 September 2025. Employing USDT and USDC as representative digital currency market variables alongside traditional financial assets including equities, bonds, and gold, the analysis utilizes multiple econometric methodologies and yields the following principal conclusions.

First, the digital currency market exhibits the quintessential characteristics of emerging assets, demonstrating high returns accompanied by high volatility. Both the mean and standard deviation of digital currency trading volume returns substantially exceed those of traditional financial markets, aligning with the typical attributes of nascent alternative assets. Under normal market conditions, digital currency and traditional financial markets display weak and unstable static correlations, with relatively low cross-market correlations and even mild counter-cyclical movement patterns, suggesting that digital currencies offer certain portfolio diversification benefits. Conversely, traditional financial markets exhibit stable internal linkage mechanisms, with correlations among equities, bonds, and gold proving statistically significant and economically intuitive.

Second, asymmetric yet weak bidirectional dynamic risk transmission exists between digital currency and traditional financial markets. Traditional financial markets, particularly equity markets, exert somewhat more noticeable contemporaneous or near-term effects on digital currency markets, yet these effects are transient and do not constitute a stable predictive relationship—consistent with the non-significant Granger causality findings. Meanwhile, volatility in digital currency markets is primarily driven by endogenous factors, with spillover effects to traditional markets remaining limited, positioning digital currency markets largely as volatility recipients.

Third, extreme events structurally reshape cross-market correlations, with digital currency markets demonstrating exceptional sensitivity to macroeconomic policy shocks such as the Federal Reserve’s aggressive rate hikes. During extreme events, cross-market correlations intensify significantly and may even experience directional reversals. Established correlation patterns within traditional financial markets may also be disrupted under extreme scenarios, with liquidity shocks emerging as critical factors triggering cross-market risk comovement. Different categories of extreme events exhibit heterogeneous impacts: macroeconomic policy events exert the highest overall impact intensity on the financial system, financial crisis events demonstrate the broadest scope of influence, while geopolitical conflict events produce more structurally targeted effects.

Fourth, dynamic correlations between digital assets and traditional assets exhibit pronounced instability and state dependence. Extreme risk events serve as critical catalysts driving structural breaks in correlations, with market linkage patterns undergoing regime switching in response to evolving macro-financial conditions.

### 5.2. Policy Recommendations

#### 5.2.1. Recommendations for Regulatory Authorities

Regulatory authorities should establish comprehensive cross-market risk monitoring frameworks. Given the crucial intermediary role of stablecoins in facilitating cross-market capital flows, particular attention should be directed toward monitoring stablecoin trading volume dynamics as key indicators for observing cross-market capital movements and risk transmission channels. Concurrently, real-time surveillance of linkages between digital currency markets and traditional equity and bond markets should be strengthened, with special focus on correlation changes during extreme scenarios including macroeconomic policy adjustments, financial crises, and geopolitical conflicts to mitigate cross-market contagion risks. Advanced analytical techniques including big data and artificial intelligence should be employed to optimize monitoring models and enhance predictive capabilities regarding risk spillover pathways and intensity.

Macroprudential supervision of digital currency markets should be reinforced. Given the pronounced sensitivity of digital currency markets to monetary policy adjustments, policymakers should fully consider potential spillover effects on digital currency markets when formulating and implementing macroeconomic policies to avoid triggering excessive market volatility. Comprehensive regulatory frameworks governing stablecoin issuance and trading should be developed, establishing clear capital adequacy requirements, liquidity management standards, and disclosure obligations for issuing entities to mitigate cross-market disruptions arising from stablecoin liquidity risks. Regulatory oversight of institutional investor participation in digital currency markets should be enhanced, with leveraged trading activities subject to appropriate restrictions to curb excessive speculation.

International regulatory coordination and standard harmonization should be strengthened. Given the cross-border nature of digital currency markets and the global transmission of extreme event risks, enhanced international regulatory cooperation is imperative, with efforts directed toward establishing unified regulatory standards and risk management mechanisms. Active participation in global crypto-asset regulatory standard-setting processes should be pursued, promoting international collaboration in areas including stablecoin regulation, cross-border capital flow monitoring, and risk information sharing. Cross-border risk early warning and emergency response mechanisms should be established to collectively mitigate systemic financial risk contagion across borders.

#### 5.2.2. Recommendations for Investors

Investors should exercise prudent judgment in allocating digital currency assets. Based on the weak correlation characteristics between digital currency and traditional financial markets, investors may consider incorporating digital currencies as emerging asset classes into portfolio construction to enhance diversification benefits. However, full recognition of the high-volatility risk profile is essential, with allocation proportions appropriately calibrated according to individual risk tolerance levels to avoid excessive speculation. Attention should be directed toward stablecoin trading volume dynamics as signals reflecting cross-market capital flows, optimizing the timing of portfolio adjustments.

The impact of extreme events on portfolio performance warrants careful consideration. During extreme events, correlations between digital currency and traditional assets may intensify significantly, potentially diminishing diversification benefits. Under such circumstances, investors should promptly adjust portfolio allocations to reduce risk exposure. Differentiated asset allocation strategies should be formulated in response to heterogeneous impacts of various extreme event types. For instance, during monetary policy tightening periods, appropriate reduction in digital currency and equity allocations may be warranted, while geopolitical conflict periods may support moderately increasing allocations to traditional safe-haven assets such as gold.

Liquidity risk management deserves particular emphasis. Under extreme scenarios, liquidity shocks may trigger simultaneous selling pressure across multiple asset classes. Investors should maintain prudent capital structure arrangements, avoiding excessive leverage while ensuring sufficient portfolio liquidity to withstand market volatility. Investment should be directed toward digital currency assets with robust liquidity characteristics to minimize transaction costs and liquidation risks under extreme market conditions.

## Figures and Tables

**Table 1:** Empirical variable processing and economic interpretation.

Variable Category	Variable Name	Variable Symbol	Calculation Method	Economic Interpretation
Equity Market	S&P 500 Index	*sp500*	Logarithmic difference in daily closing prices	Reflects the overall performance of the US stock market
Bond Market	10-year Treasury Bond Yield	*bond*	Logarithmic difference in daily closing yields	Reflects market expectations for economic growth and inflation
Commodity	Gold Futures Price	*gold*	Logarithmic difference in daily closing prices	Reflects market safe-haven sentiment and inflation expectations
Digital Currency	Trading Volume	*volume*	Logarithmic difference in total daily trading volume (USDT + USDC)	Reflects overall activity and liquidity changes in the digital currency market
Volatility Indicator	VIX Volatility Index	*vix*	Logarithmic difference in daily closing prices	Reflects market expectations for volatility over the next 30 days
Exchange Rate Market	US Dollar Index	*index*	Logarithmic difference in daily closing prices	Reflects the overall strength of the US dollar

**Table 2:** Descriptive statistics and stationarity test results.

Variable	Mean	Std. Dev.	Skewness	Kurtosis	Jarque–Bera Test (*p*-Value)	ADF Statistic (*p*-Value)	Stationarity Conclusion
*volume*	0.001441	0.318469	0.433685	3.328669	586.69 (0.0000)	−46.79 (0.0100)	Stationary
*sp500*	0.000497	0.010803	0.012684	6.384117	2020.90 (0.0000)	−35.14 (0.0100)	Stationary
*bond*	0.001246	0.022585	0.111198	1.906229	181.70 (0.0000)	−34.89 (0.0100)	Stationary
*gold*	0.000578	0.009814	−0.35998	1.575503	148.78 (0.0000)	−35.49 (0.0100)	Stationary
*vix*	−0.00042	0.077191	1.095369	7.770261	3231.66 (0.0000)	−36.72 (0.0100)	Stationary
*index*	0.000071	0.004523	−0.31384	1.508608	132.38 (0.0000)	−34.37 (0.0100)	Stationary

**Table 3:** Correlation coefficient matrix.

Variable	*volume*	*sp500*	*bond*	*gold*	*vix*	*index*
*volume*	1.0000					
*sp500*	−0.0751 *** (−2.60)	1.0000				
*bond*	−0.0424 (−1.46)	−0.0200 (−0.69)	1.0000			
*gold*	−0.0277 (−0.95)	0.0651 (2.25)	−0.2802 *** (−10.03)	1.0000		
*vix*	0.1300 *** (4.52)	−0.7643 *** (−40.85)	−0.0466 (−1.60)	−0.0183 (−0.63)	1.0000	
*index*	0.0149 (0.51)	−0.2480 *** (−8.82)	0.2429 *** (8.61)	−0.4477 *** (−17.26)	0.1563 *** (5.45)	1.0000

Note: t-statistics are presented in parentheses. *** denote statistical significance at the 1% levels.

**Table 4:** VAR system test results.

Diagnostic Type	Test Item	Statistic/Value	Diagnostic Conclusion
Stability Test	Maximum Modulus of Characteristic Roots	0.2975	System is stable (<1)
Residual Autocorrelation	Portmanteau Q Test	0.5488 (*p* = 1.0000)	No significant autocorrelation
Residual Heteroskedasticity	ARCH-LM Test (Lag 5)	*p* < 0.05 for all variables	ARCH effect exists

**Table 5:** Granger causality test results (optimal lag order *p* = 1).

Null Hypothesis (H_0_)	F-Statistic	*p*-Value	Test Conclusion (at 5% Level)
S&P 500 does not Granger-cause Digital Currency Trading Volume	0.9409	0.6678	Fail to reject H_0_
Bond Yields do not Granger-cause Digital Currency Trading Volume	0.2369	0.3734	Fail to reject H_0_
Gold does not Granger-cause Digital Currency Trading Volume	0.7054	0.5989	Fail to reject H_0_
Digital Currency Trading Volume does not Granger-cause S&P 500	2.1994	0.8617	Fail to reject H_0_
Bond Yields do not Granger-cause S&P 500	4.6196	0.9682	Fail to reject H_0_
Gold does not Granger-cause S&P 500	8.6295	0.9966	Fail to reject H_0_
Digital Currency Trading Volume does not Granger-cause Bond Yields	0.6600	0.5833	Fail to reject H_0_
S&P 500 does not Granger-cause Bond Yields	0.1057	0.2548	Fail to reject H_0_
Gold does not Granger-cause Bond Yields	5.9656	0.9853	Fail to reject H_0_
Digital Currency Trading Volume does not Granger-cause Gold	0.8653	0.6476	Fail to reject H_0_
S&P 500 does not Granger-cause Gold	0.0806	0.2234	Fail to reject H_0_
Bond Yields do not Granger-cause Gold	0.8089	0.6314	Fail to reject H_0_

(Note: Some GC results among traditional assets are insignificant, which is consistent with the partial decoupling of pricing logic among assets during the sample period. Due to space limitations, the table focuses primarily on digital currency.)

**Table 6:** Variance decomposition results.

Source of Shock	Period 1	Period 2	Period 3	Period 4
Digital Currency Trading Volume	99.535%	95.155%	82.099%	90.529%
S&P 500	0.264%	4.655%	2.991%	3.955%
Bonds	0.016%	0.016%	14.750%	0.218%
Gold	0.185%	0.174%	0.159%	5.298%
Total	100%	100%	100%	100%

**Table 7:** Correlation coefficient differences between normal and extreme periods.

Variable Relationship	Normal Period	Extreme Period	Difference	Z Statistic	*p*-Value
volume-sp500	−0.0719	−0.1105	−0.0385	0.2839	0.7765
volume-bond	−0.064	0.2161	0.2801	−2.0714	0.0383
volume-gold	−0.019	−0.1861	−0.167	1.2360	0.2165
sp500-bond	−0.0056	−0.1891	−0.1835	1.3573	0.1747
sp500-gold	0.0561	0.2204	0.1643	−1.2262	0.2201
bond-gold	−0.2738	−0.4293	−0.1555	1.3006	0.1934

**Table 8:** Definition of extreme risk event periods.

Event Name	Start Date	End Date	Trading Days
Suez Canal Blockage	2 March 2021	14 April 2021	31
Russia–Ukraine Conflict	2 February 2022	17 March 2022	31
Fed Aggressive Rate Hikes	24 May 2022	8 July 2022	31
RCEP Takes Effect	9 November 2022	22 December 2022	31
Silicon Valley Bank Collapse	16 February 2023	31 March 2023	31
Israel–Hamas Conflict	15 September 2023	27 October 2023	31
U.S. Tariff Hikes	11 July 2024	22 August 2024	31
Trump Inauguration	26 December 2024	11 February 2025	31
UN Lowers GDP Forecast	9 April 2025	22 May 2025	31

Note: Each event window is centered on the event date, extending 15 trading days before and after, for a total of 31 trading days (including the event day). This design captures short-term market reactions around each event while maintaining consistent window lengths for comparable cross-event analysis.

**Table 9:** Robustness test results.

Variable Relationship	Subsample 1 (First 50%) Correlation	Subsample 2 (Second 50%) Correlation	Difference
volume-sp500	−0.1201	−0.0171	0.1030
volume-bond	−0.0321	−0.0695	−0.0374
volume-gold	0.0124	−0.0702	−0.0826
sp500-bond	−0.0044	−0.056	−0.0516
sp500-gold	0.079	0.0464	−0.0326
bond-gold	−0.3266	−0.2355	0.0911

## Data Availability

The data supporting the plots within this paper and other studies. Findings are available from the corresponding author upon reasonable request.
